# Hepatitis B in Hemodialysis: Serologic Dynamics and Implications for Care

**DOI:** 10.3390/jcm15082950

**Published:** 2026-04-13

**Authors:** Rawi Hazzan, Nana Peleg, Tarek Saadi, Mahmood Mahajna, Maanit Shapira, Yana Tal, Ahlam Bsoul, Oren Gal, Fadi Abu Baker

**Affiliations:** 1 Clalit Health Services, Nof Hagalil 1766201, Israel; 2 Department of Nephrology, Hillel Yaffe Medical Center, Hadera 38100, Israel; 3 Center for Liver Diseases, Rambam Healthcare Campus, Haifa 3109601, Israel; 4 Department of Clinical Pharmacy, Faculty of Medicine, Ben-Gurion University of the Negev, Beersheva 8410501, Israel; 5 Pharmacology Department, Hillel Yaffe Medical Center, Hadera 38100, Israel; 6 Laboratory Division, Hillel Yaffe Medical Center, Hadera 38100, Israel; 7 Department of Gastroenterology and Hepatology, Hillel Yaffe Medical Center, Hadera 38100, Israel; 8 Bruce Rappaport Faculty of Medicine, Technion—Israel Institute of Technology, Haifa 3525433, Israel

**Keywords:** hepatitis B virus, hemodialysis, vaccination, occult HBV infection, waning immunity, booster response, serologic monitoring, chronic kidney disease

## Abstract

**Background**: Hemodialysis patients are particularly vulnerable to hepatitis B virus (HBV) due to immunosuppression and repeated vascular access. While universal childhood vaccination has reduced population-level HBV prevalence, dialysis units require tailored prevention and monitoring strategies. This study aimed to characterize HBV serologic profiles, evaluate immune responses, and assess the kinetics of antibody waning in a diverse hemodialysis population. **Methods**: We retrospectively analyzed 565 adult hemodialysis patients (2015–2024), assessing HBV seroprevalence, seroconversion, booster response, and antibody waning. Subgroup comparisons were made by ethnicity and birth cohort (pre- vs. post-1992 national vaccine rollout). Time-to-waning analyses were performed using Kaplan–Meier methods. **Results**: HBsAg and anti-HBc were positive in 4.1% and 31.7% of patients, respectively; 3.7% were HCV seropositive. No HBsAg seroconversions occurred, and 2.1% of initially anti-HBc-negative patients seroconverted. Among patients with isolated anti-HBc, 80.9% developed protective anti-HBs titers, and none became HBsAg- or HBV DNA-positive. Waning anti-HBs titers occurred in 67.5% (median: 7.3 months), with 87.4% demonstrating a serologic response following documented vaccine delivery. Patients born after 1992 showed higher isolated anti-HBs positivity and lower anti-HBc prevalence. Ethnic subgroup analysis showed higher exposure rates but similar booster response among minority patients. **Conclusions**: HBV serologic profiles in this hemodialysis cohort reflected the interplay of immunosuppression, vaccination practices, and evolving epidemiologic trends. Subgroups exhibited variable vaccine responses, differing patterns of antibody waning, and a low incidence of new infections. These findings support tailored, population-specific HBV monitoring and prevention strategies in dialysis care.

## 1. Introduction

Hepatitis B virus (HBV) infection remains a significant global public health concern, particularly among vulnerable populations such as individuals receiving maintenance hemodialysis [1,2]. While the widespread implementation of universal HBV vaccination has led to a substantial decline in infection rates across many regions, dialysis patients continue to face heightened risks of HBV exposure and transmission due to repeated vascular access, potential for nosocomial spread, and a reduced immunologic response to vaccination [3,4].

In dialysis units, stringent infection control protocols and routine serologic screening are essential for minimizing HBV transmission risk. In recent years, comprehensive HBV screening at dialysis initiation, including HBsAg, anti-HBs, and anti-HBc, has been increasingly adopted in many healthcare systems, although their global implementation remains inconsistent. Regular monitoring of these markers is especially critical for detecting waning vaccine-induced immunity and identifying serologic patterns suggestive of prior exposure, nonresponse to vaccination, or occult infection [5,6]. In particular, individuals with anti-HBc positivity in the absence of both HBsAg and anti-HBs—termed isolated anti-HBc positivity—pose unique diagnostic and clinical challenges [7].

The interpretation of isolated anti-HBc positivity in dialysis patients is complicated by its potential to represent resolved infection, occult HBV infection (OBI), or biological false reactivity. Although several studies suggest that the prevalence of OBI in this population is low, its existence remains clinically relevant, particularly in immunocompromised individuals, where HBV reactivation poses significant risks [8]. Current guidelines vary in their approach to managing isolated anti-HBc positivity, and high-quality evidence guiding vaccination or surveillance strategies in this subgroup is lacking [9].

In parallel, there is increasing recognition of the phenomenon of waning HBV immunity in long-term dialysis patients. Declining anti-HBs titers after initial vaccination or booster dosing may reduce seroprotection, necessitating ongoing monitoring and timely revaccination. While annual anti-HBs testing is widely recommended, the optimal timing and clinical impact of such declines remain poorly defined. Moreover, evolving vaccination strategies—including initiating HBV vaccination earlier in the course of chronic kidney disease (CKD), prior to dialysis dependence—have further altered the serologic landscape and necessitate updated real-world data to guide care [10].

From a health policy perspective, these clinical uncertainties translate into inconsistent national practices, potential gaps in protective immunity, and resource allocation challenges for dialysis providers and public health authorities. Decisions regarding the frequency of serologic monitoring, use of booster vaccination, and interpretation of isolated anti-HBc results have important implications for cost-effective, equitable, and evidence-based HBV prevention strategies. Furthermore, differences in HBV serologic patterns by ethnicity or birth cohort may reflect underlying disparities in vaccine access, health literacy, or care delivery—areas of central concern for public health planning and policy [11].

This study aimed to evaluate the serologic dynamics of HBV in a diverse cohort of patients receiving maintenance hemodialysis to inform clinical and public health approaches to infection control, vaccination, and serologic surveillance in this high-risk population.

## 2. Methods

### 2.1. Study Design and Setting

This retrospective cohort study was conducted at the dialysis unit of Hillel Yaffe Medical Center, a university-affiliated hospital in Israel. It included all patients undergoing maintenance hemodialysis between 2015 and 2024 who had available HBV serologic testing recorded in their electronic health records during this period. During the study period, a total of 582 patients receiving maintenance hemodialysis underwent HBV serologic testing at our center. The study setting reflects a standard dialysis delivery model within Israel’s public health system, allowing findings to inform national infection control strategies and vaccination policies. The study was approved by the institutional ethics committee of Hillel Yaffe Medical Center, which also granted a waiver of informed consent due to its retrospective and non-interventional nature. Patient confidentiality was maintained throughout by the Declaration of Helsinki.

### 2.2. Study Population and Data Collection

The analytic cohort included adult patients (age ≥ 18 years) receiving chronic maintenance hemodialysis who had, at baseline, at least one full set of hepatitis B serologic markers—hepatitis B surface antigen (HBsAg), antibody to hepatitis B surface antigen (anti-HBs), and antibody to hepatitis B core antigen (anti-HBc). Patients were followed longitudinally, and repeat serologic testing was performed as part of routine clinical care.

Patients were excluded if they had known background conditions that could significantly alter the natural course or management of HBV infection, interfere with follow-up, or represent special populations with distinct immunological profiles. These included individuals with cirrhosis, advanced malignancy or ongoing chemotherapy, biologic immunosuppressive therapy, known immunodeficiency syndromes, or HIV infection. In addition, patients were excluded if they lacked a complete baseline HBV serologic profile or had insufficient longitudinal serologic follow-up, defined as fewer than two HBV serologic assessments separated by at least 12 months.

Demographic data (age, sex, ethnicity), clinical background, and longitudinal laboratory results were extracted. HBV serologic markers (HBsAg, anti-HBs, and anti-HBc) were collected at baseline and serially throughout the follow-up period. HBV serologic testing included hepatitis B surface antigen (HBsAg), antibody to hepatitis B surface antigen (anti-HBs), and total antibody to hepatitis B core antigen (anti-HBc). HBsAg, anti-HBs, and total anti-HBc were measured using electrochemiluminescence immunoassays (Elecsys HBsAg II, Anti-HBs II, and Anti-HBc II; Roche Diagnostics, Mannheim, Germany) on cobas e analyzers. Anti-HBs titers were quantified in mIU/mL, with levels ≥10 mIU/mL considered protective. Anti-HBc testing reflected total antibodies; anti-HBc IgM testing was performed selectively in HBsAg-positive patients when clinically indicated to distinguish acute from chronic infection.

HBV DNA was quantified using the Xpert^®^ HBV Viral Load real-time PCR assay (Cepheid, Sunnyvale, CA, USA), with a lower limit of quantification of 10 IU/mL. HBV DNA testing was performed in all HBsAg-positive patients and in those with anti-HBc positivity, with or without anti-HBs, and when clinically indicated during follow-up. Anti-HCV and HCV RNA results were also recorded when available. Data on the timing of HBV vaccination were retrieved when available; however, information on vaccine formulation was inconsistently documented and, therefore, not systematically included in the analysis.

### 2.3. Serologic Definitions and Outcomes

Patients were classified according to their HBV serologic profiles: HBsAg positivity was considered indicative of active or chronic infection; anti-HBc positivity reflected prior exposure (either resolved or ongoing); and anti-HBs positivity was interpreted as evidence of immunity, either vaccine-induced or post-infection. Information on hepatitis B vaccination and revaccination was obtained from documented vaccine delivery records in the electronic medical record, which in routine dialysis practice are used to document vaccine administration. Exact injection timestamps and vaccine formulation details were not uniformly available.

Isolated anti-HBc was defined as anti-HBc positivity in the absence of both HBsAg and anti-HBs. The primary outcomes included the prevalence and temporal dynamics of HBV Sero markers, the rate of new HBV exposure or seroconversion, and the incidence of HBsAg seroconversion. Among anti-HBc-positive, HBsAg-negative patients, anti-HBs titers were monitored to assess response to revaccination. Waning immunity was defined as the first observed decline in anti-HBs titers from ≥10 mIU/mL to <10 mIU/mL during follow-up. Subgroup analyses were conducted based on ethnicity (Jewish vs. Arab) and birth cohort (pre- vs. post-1992), to evaluate the influence of national vaccination implementation. In patients with waning immunity, the time to antibody loss was assessed using Kaplan–Meier survival analysis.

### 2.4. Statistical Analysis

Descriptive statistics were used to summarize baseline characteristics. Categorical variables were expressed as frequencies and percentages, while continuous variables were reported as means ± standard deviations or medians with interquartile ranges, as appropriate. Group comparisons were made using the chi-square or Fisher’s exact test for categorical variables and the Student’s *t*-test or Mann–Whitney U test for continuous variables. Waning immunity was analyzed using time-to-event methods. The median time to loss of protective anti-HBs levels was estimated using the Kaplan–Meier analysis. *p*-values < 0.05 were considered statistically significant. All analyses were conducted using SPSS Statistics version 27 (IBM Corp., Armonk, NY, USA).

## 3. Results

### 3.1. Cohort Characteristics

Of the 582 patients undergoing maintenance hemodialysis who had HBV serologic testing during the study period, 565 met the inclusion criteria and constituted the final analytic cohort, contributing over 3000 serologic test results during longitudinal follow-up. The cohort comprised 327 males (57.9%) and 238 females (42.1%). With respect to ethnicity, 384 patients (68.0%) were classified as Jewish, and 181 patients (32.0%) were classified as Arab. The median follow-up duration was 1048 days (approximately 34.5 months), with an interquartile range of 463 to 1645 days and a range from 297 to 3428 days.

**Figure 1:**
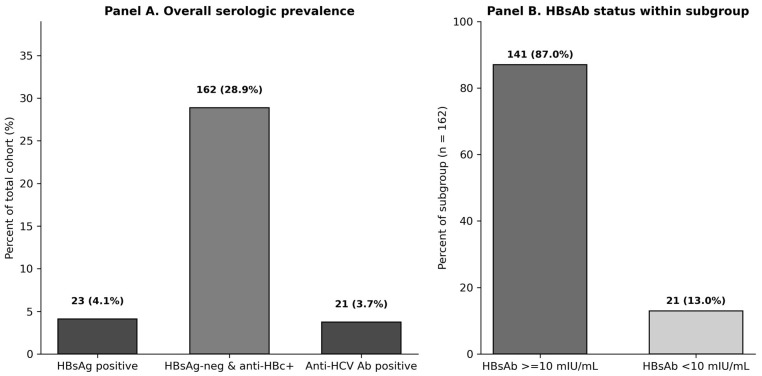
Distribution of hepatitis B and C serologic profiles among the study cohort. Panel (**A**) illustrates the prevalence of key HBV and HCV serologic markers in the overall cohort, including HBsAg positivity at any time, HBsAg-negative/anti-HBc-positive status, and anti-HCV antibody positivity. Panel (**B**) depicts hepatitis B surface antibody (HBsAb) levels among HBsAg-negative, anti-HBc-positive patients (*n* = 162), stratified by protective (≥10 mIU/mL) and non-protective (<10 mIU/mL) titers. Abbreviations: HBsAg—hepatitis B surface antigen; anti-HBc—hepatitis B core antibody; HBsAb—hepatitis B surface antibody; HCV—hepatitis C virus.

Across all time points, 23 patients (4.1%) tested positive for HBsAg, consistent with active or chronic HBV infection (Figure 1). Anti-HBc was positive in 179 patients (31.7%), indicating active or prior exposure. Notably, among the 386 patients who were initially anti-HBc-negative, a total of 8 patients (2.1%) developed anti-HBc positivity during follow-up, indicating new HBV exposure or seroconversion over time. Of the anti-HBc positive patients, 162 were HBsAg negative. Among HBsAg negative, anti-HBc-positive patients, 21 (12.9%) had anti-HBs (HBsAb) levels below the protective threshold of 10 mIU/mL at baseline, while 141 (87.1%) had protective HBsAb titers ≥ 10 mIU/mL. All of these patients had undetectable HBV DNA at baseline.

Anti-HCV antibodies (HCVAb) were detected in 21 patients (3.7%) over the study period. Among them, 6 patients had detectable HCV RNA, confirming chronic HCV infection. Three of these 6 individuals were also anti-HBc-positive; none had HBsAg positivity.

### 3.2. Serologic Profiles by Ethnicity

Serologic profiles were stratified by ethnicity to assess patterns of HBV and HCV exposure and immunity (Table 1). Anti-HBc positivity was observed in 117 of 384 Jewish patients (30.5%) and in 62 of 181 Arab patients (34.3%). HBsAg positivity was documented in 13 Jewish (3.4%) and 10 Arab patients (5.5%). The proportion of patients with isolated HBsAb positivity (HBsAg-negative, anti-HBc-negative, HBsAb ≥ 10 mIU/mL) was numerically higher among Jewish patients compared with Arab patients; however, this difference did not reach statistical significance (33.6% vs. 28.7%, *p* = 0.289). In contrast, the overall rate of serologic markers indicating current or past HBV infection (HBsAg and/or anti-HBc positivity) was higher in Arab patients. The rate of HBsAg seroconversion during follow-up among initially HBsAg-negative patients was zero in both ethnic groups.

Regarding HCV serology, anti-HCV antibodies were detected in 16 Jewish patients (4.1%) and 5 Arab patients (2.8%) (*p* = 0.435), suggesting a trend toward higher HCV seropositivity among Jewish patients. No statistically significant differences in HCVAb prevalence were observed between ethnic groups.

### 3.3. Seroconversion and Vaccination Response in Anti-HBc-Positive Patients

Among the 162 anti-HBc-positive, HBsAg-negative patients who underwent HBV DNA testing, no detectable viremia was identified. Similarly, no HBsAg seroconversions were observed among patients who experienced waning anti-HBs titers during follow-up. This included patients with low baseline HBsAb levels and those with protective titers. Among the 21 patients who were anti-HBc-positive, HBsAg-negative, and had HBsAb < 10 mIU/mL at baseline, 17 (80.9%) achieved serologic response with HBsAb levels ≥ 10 mIU/mL during follow-up, consistent with a serologic response observed following documented vaccine delivery in routine clinical practice. Among the 141 anti-HBc-positive patients with HBsAb ≥ 10 mIU/mL at baseline, none developed HBsAg positivity over time. However, substantial variability in HBsAb titers was observed, and many exhibited waning immunity, defined as a subsequent drop in HBsAb to <10 mIU/mL.

### 3.4. Waning Immunity and Booster Response

Among all patients with initial HBsAb titers ≥ 10 mIU/mL (*n* = 354), 239 (67.5%) experienced a decline in antibody levels below the protective threshold during follow-up. Among these, 209 patients (87.4%) demonstrated a serologic response following documented vaccine delivery in routine clinical practice, while 30 (12.6%) failed to reattain protective titers.

**Figure 2:**
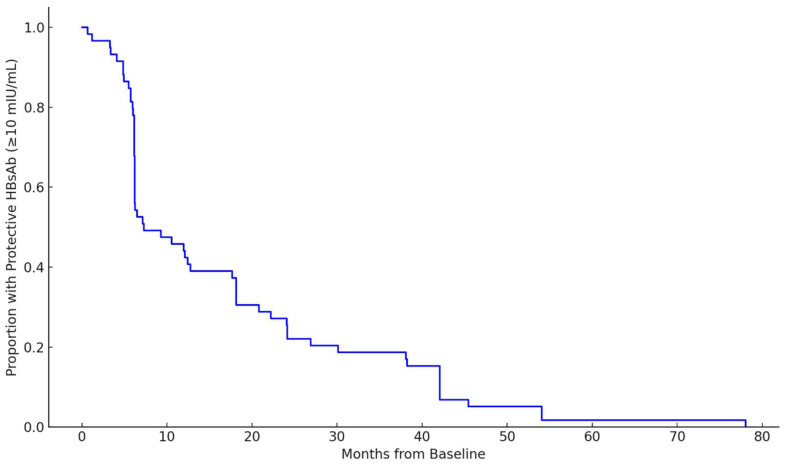
Time to lose protective anti-HBs titers in patients with baseline HBsAb ≥ 10 mIU/mL. The Kaplan–Meier survival curve demonstrates the proportion of patients maintaining protective anti-HBs titers (≥10 mIU/mL) over time following baseline testing. The most pronounced decline occurred within the first 6–12 months, with a median time to loss of seroprotection of 7.3 months (IQR: 6.2–24.1 months). Abbreviations: HBsAb—hepatitis B surface antibody; IQR—interquartile range.

A Kaplan–Meier-style curve was constructed to evaluate the temporal dynamics of waning immunity (Figure 2). The most pronounced decline in HBsAb was observed within the first 6 to 12 months following baseline. The median time to loss of protective HBsAb titers was 7.3 months (interquartile range: 6.2–24.1 months). No significant differences in time to antibody loss or booster response were observed by sex or ethnicity.

### 3.5. Serologic Profiles by Birth Cohort

**Figure 3:**
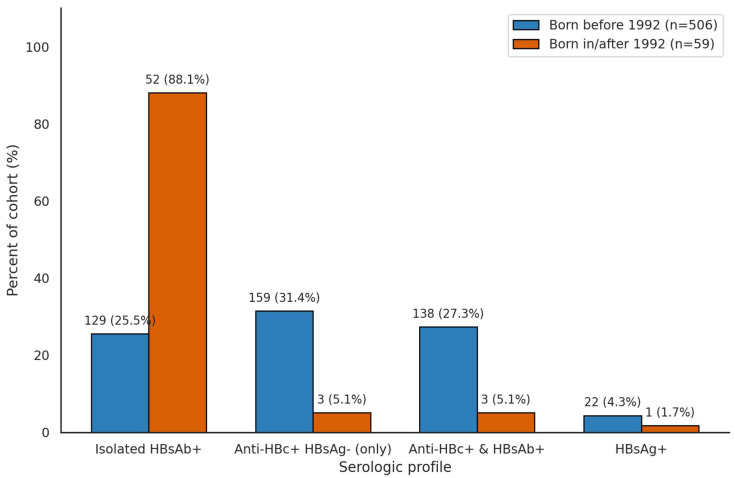
Hepatitis B serologic profiles stratified by birth cohort (Before vs. After 1992). Stacked bar chart comparing HBV serologic profiles between patients born before 1992 and those born after the implementation of the national hepatitis B vaccination program in Israel. Notable differences were observed in the prevalence of isolated HBsAb positivity (HBsAg-negative, Anti-HBc-negative, HBsAb-positive), which was significantly higher in the post-1992 cohort, and in the rates of anti-HBc and HBsAg positivity, which were markedly more common in patients born before 1992. Abbreviations: HBsAg—hepatitis B surface antigen; Anti-HBc—hepatitis B core antibody; HBsAb—hepatitis B surface antibody.

Serologic markers were compared between patients born before 1992 (*n* = 506) and those born in or after 1992 (*n* = 59), reflecting pre- and post-implementation of the national hepatitis B vaccination program. Among patients born after 1992, 52 (88.1%) exhibited isolated HBsAb positivity, compared to 129 (25.5%) in the pre-1992 cohort. Anti-HBc-positive, HBsAg-negative profiles were observed in 159 (31.4%) of those born before 1992 and only 3 (5.1%) in the post-1992 group. Similarly, anti-HBc-positive with concurrent HBsAb-positive serology was present in 138 (27.3%) of the pre-1992 cohort and 3 (5.1%) of the post-1992 cohort. HBsAg positivity remained low in both groups: 22 patients (4.3%) born before 1992 and 1 patient (1.7%) born after 1992. These patterns are illustrated in Figure 3.

## 4. Discussion

Patients receiving maintenance hemodialysis represent a uniquely immunocompromised population, characterized by impaired humoral and cellular immune responses. This immunodeficiency not only predisposes them to a higher risk of infections but also affects their response to vaccinations, including those against HBV [12,13]. Our cohort observed an HBsAg positivity rate of 4.1% and an anti-HBc positivity rate of 31.7%. This HBsAg prevalence aligns with the lower end of estimates reported in developed regions. A recent global meta-analysis by Kahlesi et al. reported pooled HBsAg prevalence rates of 4.32% in North America and 7.07% in Europe among hemodialysis patients, underscoring the ongoing burden of HBV infection even in well-resourced settings [14]. Moreover, these figures are markedly higher than the general Israeli population’s HBV prevalence, highlighting the heightened vulnerability of dialysis patients to HBV infection [15].

A stratified analysis revealed a trend toward higher HBV seropositivity among Arab patients compared to their Jewish counterparts. Specifically, HBsAg positivity was documented in 5.5% of Arab patients versus 3.4% of Jewish patients, and anti-HBc positivity was observed in 34.3% of Arabs compared to 30.5% of Jews. These findings align with previous studies indicating that certain ethnic groups, including Arabs, may have higher baseline HBV prevalence due to various demographic, socio-economic, and cultural factors [16].

Despite the elevated baseline prevalence, the incidence of new HBV infections during the study period was remarkably low, with only 2.1% of initially anti-HBc-negative patients seroconverting to anti-HBc-positive status. This low seroconversion rate may reflect effective infection control practices within dialysis units, including strict adherence to hygiene protocols, patient isolation when necessary, and routine screening. Notably, current nephrology practice increasingly emphasizes initiating hepatitis B vaccination earlier in the course of chronic kidney disease, prior to the onset of dialysis dependence. This approach has been shown to improve seroconversion rates and durability of vaccine-induced immunity, likely due to better preserved immunologic function in earlier CKD stages [17]. In our cohort, this evolving strategy may be reflected in the significantly higher prevalence of isolated HBsAb positivity among younger patients, consistent with implementing routine childhood and pre-dialysis vaccination programs.

An intriguing subset of our cohort comprised patients who were anti-HBc-positive but negative for both HBsAg and anti-HBs at baseline. Among these individuals, 80.9% achieved serologic response with anti-HBs levels ≥10 mIU/mL during follow-up, indicating a likely response to HBV booster vaccination. Importantly, none of these patients developed detectable HBV DNA or seroconverted to HBsAg positivity throughout the observation period. This pattern suggests that a substantial proportion of isolated anti-HBc results in dialysis patients may reflect nonspecific or clinically insignificant anti-HBc reactivity rather than actual evidence of past infection or occult hepatitis B virus (HBV) infection (OBI) [18]. Nonetheless, OBI, the presence of HBV DNA in the absence of HBsAg, cannot be entirely excluded. Although no such cases were identified in our cohort, OBI remains a clinically relevant concern, particularly in patients with isolated anti-HBc positivity. This is supported by findings from Sowole et al., who reported a 2.2% prevalence of OBI among 138 anti-HBc-positive, HBsAg-negative hemodialysis patients with HBV DNA levels that were low and clinically silent [19]. These results reinforce the importance of risk-based screening strategies and suggest that while OBI is uncommon in settings with routine vaccination and robust infection control, it should still be considered in select populations. In this context, beyond HBV DNA testing, emerging serologic markers have been proposed to further refine the assessment of occult hepatitis B infection. Hepatitis B core-related antigen (HBcrAg), which reflects transcriptional activity from covalently closed circular DNA, has shown promise as a surrogate marker of low-level viral persistence in HBsAg-negative individuals. In parallel, quantitative anti-HBc (qAnti-HBc) levels have been associated with prior viral exposure and intrahepatic viral activity and may help stratify the likelihood of OBI among patients with isolated anti-HBc positivity. While these assays are not yet routinely available in clinical practice and remain primarily research tools, they may represent valuable adjuncts to HBV DNA testing for selected high-risk populations in the future [20,21].

Furthermore, the consistently low prevalence of OBI reported across multiple studies [22,23] supports the position that anti–HBc-positive, HBV DNA-negative patients should not be excluded from receiving HBV vaccination. On the contrary, our findings support a simplified and pragmatic policy approach: isolated anti-HBc positivity, in the absence of detectable DNA, should not delay or preclude revaccination. This could reduce unnecessary monitoring and support resource optimization.

The introduction of routine HBV vaccination in Israel in 1992 has significantly impacted patients’ serologic profiles. In our study, 88.1% of patients born after 1992 exhibited isolated anti-HBs positivity compared to only 25.5% of those born before 1992. This substantial difference underscores the success of the national vaccination program in reducing HBV transmission and altering the seroepidemiology of the disease, providing reassurance about the effectiveness of vaccination strategies [24].

However, waning immunity remains a significant concern in the dialysis population. Although waning of anti-HBs titers was common, no HBsAg seroconversions were observed during follow-up and anti-HBc seroconversion was rare at the cohort level. Current practice in hemodialysis relies on periodic anti-HBs surveillance and booster dosing when titers fall below 10 mIU/mL, commonly reassessed on an annual basis. In this context, our Kaplan–Meier analysis, based on the first observed decline below the protective threshold, suggests that loss of seroprotection may occur earlier than is captured by routine testing intervals. Accordingly, waning anti-HBs should be interpreted primarily as an operational signal prompting surveillance and revaccination rather than as a direct surrogate of breakthrough infection risk.

This observation highlights the relatively limited durability of serologic protection in many patients receiving dialysis, even after successful initial immunization or boosting. Encouragingly, 87.4% of patients with waning immunity demonstrated a serologic response following booster vaccination. These findings support current recommendations advocating at least annual monitoring of anti-HBs titers in patients undergoing dialysis and timely administration of booster doses when antibody levels fall below protective thresholds [25]. The data further suggest that shorter intervals for serologic reassessment, particularly within the first year following vaccination or boosting, may be warranted in select patients to ensure sustained immunity.

These findings underscore several priorities for national health policy refinement. First, integration of HBV serologic status into chronic kidney disease (CKD) and dialysis registries would enable systematic monitoring of population-level trends, support timely clinical decision-making, and facilitate outcome-based evaluation of prevention strategies. Second, the high prevalence of waning immunity supports the implementation of structured booster protocols, potentially with shortened surveillance intervals, to maintain seroprotection in this vulnerable population. Third, mandating HBV vaccination during the pre-dialysis phase—ideally at CKD stage 4—may enhance vaccine responsiveness and reduce post-initiation transmission risk. Finally, the observed differences in HBV exposure across ethnic subgroups suggest a need for targeted outreach and follow-up strategies to ensure consistent implementation of preventive measures. Together, these considerations provide a pragmatic framework for strengthening HBV control in dialysis settings through data-informed and population-responsive policy.

Our study has several limitations. It was conducted at a single center with a moderate sample size, which may limit generalizability. Vaccination timing was derived from documented vaccine delivery records rather than exact injection dates, and vaccine formulation details were not uniformly available; this may introduce minor imprecision in estimating the duration of protective immunity and in interpreting vaccine response kinetics. In addition, serologic testing and revaccination were performed as part of routine clinical care rather than at fixed protocol-defined intervals. At our center, anti-HBs titers are generally recommended to be reassessed approximately annually in vaccinated or HBV-susceptible patients, although actual testing intervals may vary in clinical practice. Accordingly, the estimated time to loss of seroprotection reflects the first observed decline below 10 mIU/mL and may be subject to interval censoring. Finally, although the follow-up period was sufficient to capture serologic dynamics and waning immunity, it may not fully reflect long-term clinical outcomes related to HBV infection. These limitations underscore the need for prospective studies with standardized vaccination and monitoring schedules to further refine HBV prevention strategies in the hemodialysis population.

## 5. Conclusions

This study demonstrates that while new HBV infections in dialysis patients are rare, waning vaccine-induced immunity is highly prevalent, underscoring the need for systematic surveillance and timely boosting. Isolated anti-HBc positivity was largely benign, supporting revaccination in the absence of detectable viremia. Marked differences between pre- and post-vaccination birth cohorts highlight the enduring success of universal vaccination. These findings argue for integrated, data-driven strategies—early vaccination in CKD, structured serologic monitoring, and targeted outreach—to sustain protective immunity and optimize HBV prevention in dialysis populations.

## Figures and Tables

**Table 1:** Baseline Characteristics and Hepatitis B and C Serologic Profiles Among the Study Cohort, Stratified by Ethnicity.

Variable	Overall (*n* = 565)	Jewish (*n* = 384)	Arab (*n* = 181)	*p*-Value
Male sex, *n* (%)	327 (57.9%)	234 (60.9%)	93 (51.4%)	0.0398
Mean age ± SD	63.08 ± 8.92	62.92 ± 9.13	63.48 ± 8.71	0.598
Age range	20–91	20–86	22–91	n/a
HBsAg-positive, *n* (%)	23 (4.1%)	13 (3.4%)	10 (5.5%)	0.136
Anti-HBc-positive, *n* (%)	179 (31.7%)	117 (30.5%)	62 (34.3%)	0.42
HCV Ab positive *n* (%)	21 (3.7%)	16 (4.1%)	5 (2.8%)	0.435
Isolated HBsAb (≥10 mIU/mL) *n* (%)	181 (32.0%)	129 (33.6%)	52 (28.7%)	0.289
Waning immunity median (months, IQR)	7.8 (6.2–24.1)	7.1 (6.2–18.2)	9.4 (6.2–19.1)	0.281

Abbreviations: HBsAg—hepatitis B surface antigen; Anti-HBc—hepatitis B core antibody; HBsAb—hepatitis B surface antibody; HCV Ab—hepatitis C virus antibody; SD—standard deviation; IQR—interquartile range. Note: *p*-values reflect comparisons between Jewish and Arab patients.

## Data Availability

The data that support the findings of this study are available on request from the corresponding author.

## List of Abbreviations

HBV	Hepatitis B virus
HBsAg	Hepatitis B surface antigen
HBsAb	Hepatitis B surface antibody
Anti-HBc	Hepatitis B core antibody
OBI	Occult hepatitis B infection
HCV	Hepatitis C virus
HCVAb	Hepatitis C virus antibody
CKD	Chronic kidney disease
PCR	Polymerase chain reaction
IQR	Interquartile range
SD	Standard deviation
IRB	Institutional Review Board
